# Virulence of *Burkholderia pseudomallei* Strains from Western Hemisphere and Africa in Mice

**DOI:** 10.3201/eid3208.260069

**Published:** 2026-08

**Authors:** Christopher P. Klimko, J. Matthew Meinig, Kevin D. Mlynek, Nathaniel O. Rill, Sergei S. Biryukov, Jennifer L. Dankmeyer, Annette M. Gray, Jennifer Chua, Stephanie A. Halasohoris, Michael L. Davies, Brian A. Smith, Carlos I. Rodriguez-Negron, Christopher T. Braun, Elsie E. Martinez, Jade L. Spencer, David N. Dyer, Ondraya M. Frick, Marjorie E. Torres, Mindy G. Elrod, Jay E. Gee, Christopher A. Gulvik, Zachary P. Weiner, Ju Qiu, Joel A. Bozue, David DeShazer, Christopher K. Cote

**Affiliations:** US Army Medical Research Institute of Infectious Diseases, Frederick, Maryland, USA (C.P. Klimko, J.M. Meinig, K.D. Mlynek, N.O. Rill, S.S. Biryukov, J.L. Dankmeyer, A.M. Gray, J. Chua, S.A. Halasohoris, M.L. Davies, B.A. Smith, C.I. Rodriguez-Negron, C.T. Braun, E.E. Martinez, J.L. Spencer, D.N. Dyer, O.M. Frick, M.E. Torres, J. Qiu, J.A. Bozue, D. DeShazer, C.K. Cote); Centers for Disease Control and Prevention, Atlanta, Georgia, USA (M.G. Elrod, J.E. Gee, C.A. Gulvik, Z.P. Weiner)

**Keywords:** *Burkholderia pseudomallei*, melioidosis, virulence, bacteria, bacterial infection, zoonoses, mice, aerosol, Western Hemisphere, Africa

## Abstract

Melioidosis, caused by *Burkholderia pseudomallei*, is an emerging disease in the United States and was declared endemic to the Gulf Coast region in 2022. Melioidosis has been sporadically reported in the American continents, the Caribbean, and more recently in Africa. We conducted lethal dose analyses in BALB/c and C57BL/6 mice exposed to small particle aerosols of *B. pseudomallei* strains from the Western Hemisphere and Africa. Those isolates exhibit a variety of virulence patterns, including rapidly-lethal disease and delayed onset of fatal disease. We found that the isolates we tested grew similarly in culture but displayed major differences in biofilm formation. Our data contribute to the growing knowledge of geographically distinct *B. pseudomallei* isolates and aid in ensuring that medical countermeasures in development are effective against a diverse collection of bacterial strains.

*Burkholderia pseudomallei*, a gram-negative facultative intracellular bacterium, causes the disease melioidosis and is critical to both public health and biodefense research communities (*1*). Historically, melioidosis is a public health concern in Southeast Asia and northern Australia; however, the global range of melioidosis is much larger than previously described (*2*–*4*). Melioidosis is highly underreported in many areas around the world, including India, Africa, the Caribbean, and the Americas (*5*–*16*). Underreporting can be attributed to extremely diverse clinical manifestations, scarcity of appropriate diagnostic tools, and a general lack of knowledge of *B. pseudomallei* by medical staff in endemic regions outside of the historically prevalent areas. However, frequent case reports detailing new clinical examples of melioidosis underscore the importance of this disease throughout the world, particularly in developing nations. *B. pseudomallei* is also considered an emerging threat in the United States because of several evolving factors: endemicity in the Gulf Coast region and the Caribbean territories of Puerto Rico and US Virgin Islands (*7*,*11*,*17*); documented fatal cases resulting from unintentional importation of the bacterium to the United States by an aromatherapy spray produced in India (*18*); evidence of locally acquired melioidosis in Texas (*12*); and recent infections in Georgia after a severe weather event (*19*).

We examined 10 phylogeographically diverse strains of *B. pseudomallei* from the Western Hemisphere and 3 strains originating from Ghana in Africa. We performed in vitro growth analyses and biofilm assays of those isolates and calculated 50% lethal dose (LD_50_) estimations in both the BALB/c and C57BL/6 mouse models of inhalational melioidosis. BALB/c mice are considered an acute model (or less resistant to *B. pseudomallei*) and are the model of choice for therapeutic medical countermeasure development (*20*). In contrast, C57BL/6 mice are considered a more chronic infection model and are the generally accepted model for vaccine development (*21*–*25*).

## Materials and Methods

### Bacterial In Vitro Growth Conditions and Assessment

We grew *B. pseudomallei *strains in 4% glycerol/1% tryp¬tone/0.5% NaCl broth for 16 hours at 37°C, shaking at 200 rpm for in vivo testing. We grew *B. pseudomallei *strains in Luria broth with 4% glycerol (LBG) to assess in vitro growth kinet¬ics. We resuspended the strains from an overnight broth culture at 37°C to a 600 nm optical density (OD_600_) of 0.2 in LBG.e resuspended the strains from an overnight broth culture at 37°C to a 600 nm optical density (OD_600_) of 0.2 in LBG. We diluted suspensions 1:10 into a 96-well microtiter plate and cultured in a Spark microplate reader (Tecan Group, https://www.tecan.com) with shaking at 37°C for ≈24 hours. We measured the OD_600_ in 15-minute intervals. We determined the actual density by subtracting the value of the respective medium-only control from the measured OD_600_.

### Biofilm Assays 

We used crystal violet staining to quantify biofilm production. We adjusted overnight *B. pseudomallei* LBG broth cultures to an OD_600_ of ≈0.2, diluted 1:10 into LBG, and then incubated at 37°C for 24 hours. Prior to staining, we measured the OD_600_ of the bacterial suspension, then aspirated the plates, washed them 3 times with phosphate buffered saline to remove planktonic cells, and fixed them with 100% ethanol for 30 minutes at room temperature (*26*). To stain the biofilm, we added 0.1% crystal violet (wt/vol) to each well for 15 min and washed 3 times with phosphate buffered saline; we then solubilized the remaining stain in 33% acetic acid. To quantify staining, we measured the OD_600_ as an indicator of biofilm formation. When necessary, we diluted the samples in 33% acetic acid to ensure that readings were within linear range. We collected >3 technical replicates for each of 4 individual repeated experiments and averaged the values for each experiment. We used control *B. pseudomallei* strains K96243 and ATS2021 as comparators to test strains with different biofilm profiles.

### Mouse Median Lethal Dose Estimations

We exposed 7–9-week-old female BALB/c or C57BL/6 mice (n = 10/group) to 5 different doses of aerosolized strains of *B. pseudomallei*. Starting-dose concentrations for each target dose in the lethal dose (LD_50_) estimate ranged from ≈10^4^ to 10^9^ colony forming units (CFU)/mL. We generated small particle aerosols (1–3 μm) by using the Biaera system (Biaera, https://www.biaera.com). We then calculated the estimated inhaled doses by using Guyton’s formula based on the CFU/mL determination of an all-glass impinger collection medium (*27*–*29*). We monitored the mice 1 time/day for animal husbandry purposes and >1 time/day for clinical scoring. Scores of 0–2 represented normal mice; scores of 3–7 indicated major clinical manifestations (e.g., less peer interaction, reduced grooming, labored breathing), and those mice warranted multiple clinical assessments per day; and final scores of >8 indicated severe clinical manifestations, and those mice were euthanized immediately. We euthanized mice in accordance with early intervention criteria. At the end of the study, we euthanized the surviving mice. We enumerated residual bacteria in lungs, spleens, and brains in select dose-groups to determine if there were any novel bacterial dissemination patterns or evidence of unusual chronicity associated with any of the previously uncharacterized strains of *B. pseudomallei*.

This research was conducted under an Institutional Animal Care and Use Committee approved protocol in compliance with the Animal Welfare Act, Public Health Service Policy on Humane Care and Use of Laboratory Animals, and other federal statutes and regulations relating to animals and experiments involving animals. The facility where this research was conducted is accredited by Association for Assessment and Accreditation of Laboratory Animal Care International and adheres to the principles stated in The Guide for the Care and Use of Laboratory Animals, National Research Council, 2011.

### Statistics

We used probit analysis to estimate the LD_50_ by using log_10_ dose as the predictor. We tested the effect of *B. pseudomallei* strain and mouse on LD_50_ by using a χ^2^ test on the basis of the probit model and under the assumption of a common effect of log_10_ dose. We estimated median time-to-death (TTD) or euthanasia and accompanying confidence limits by using Kaplan-Meier survival methods. We performed comparisons between *B. pseudomallei* strains by using Wald tests of least squared mean differences on the basis of a log-normal accelerated failure time model. We calculated the Pearson and Spearman rank-order correlation analysis of the geometric mean of biofilm versus log of LD_50_. We compared pairwise treatment groups by using linear mixed effects model for biofilm comparisons. We adjusted the multiplicity by using the Tukey method. We implemented analysis by using SAS version 9.4 (SAS Institute Inc., https://www.sas.com).

## Results

### In Vitro Growth and Biofilm Analyses

**Figure 1:**
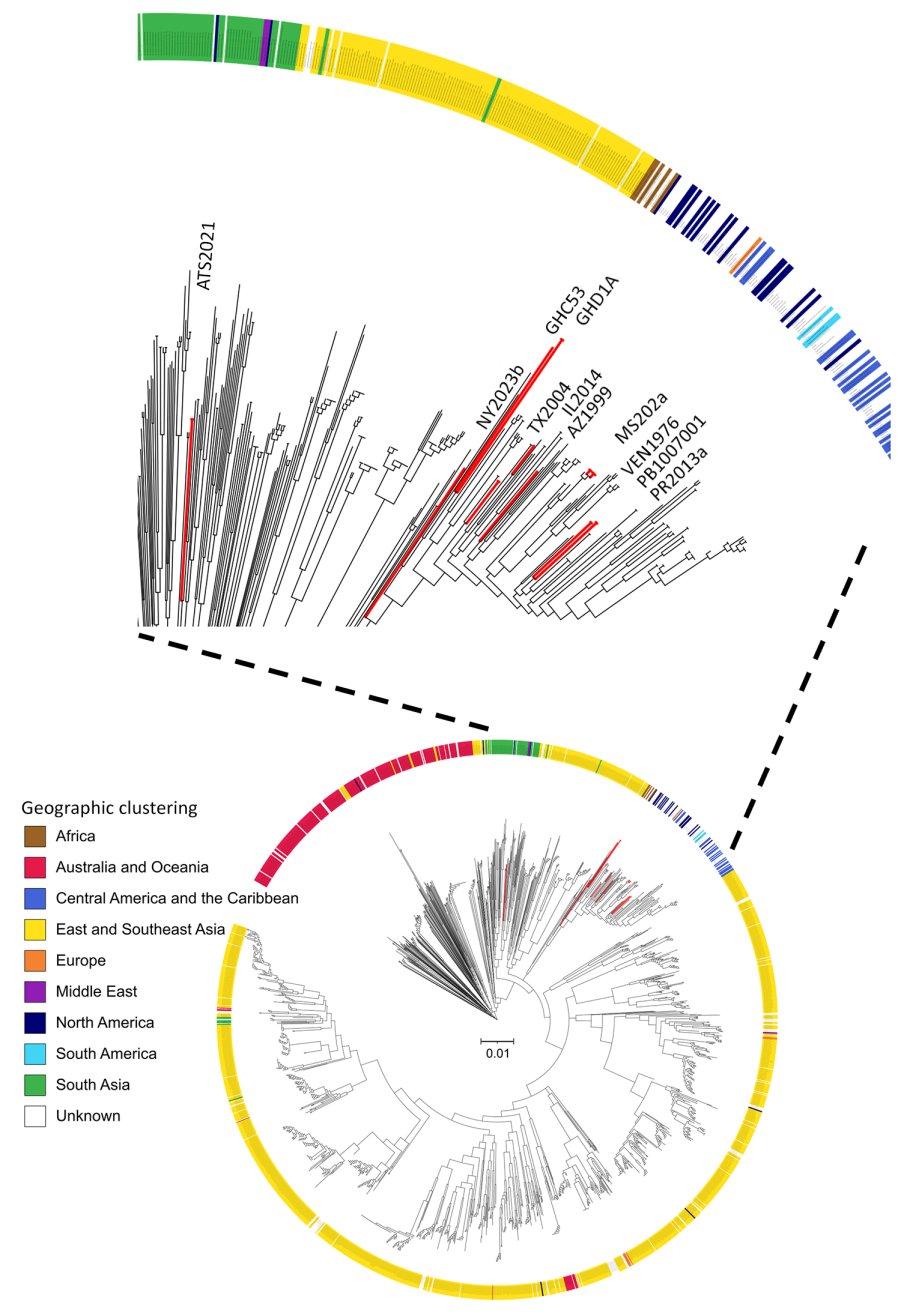
Maximum-likelihood core-genome phylogenetic tree of all *Burkholderia pseudomallei* genomes (n = 1,956) from the National Center for Biotechnology Information RefSeq (https://www.ncbi.nlm.nih.gov/refseq) database used in study of *B. pseudomallei* virulence in mouse models. Red bars highlight the 10 isolates used in this study (inset); ring color denotes geographic origin. Scale bar represents nucleotide substitutions per site.

**Figure 2:**
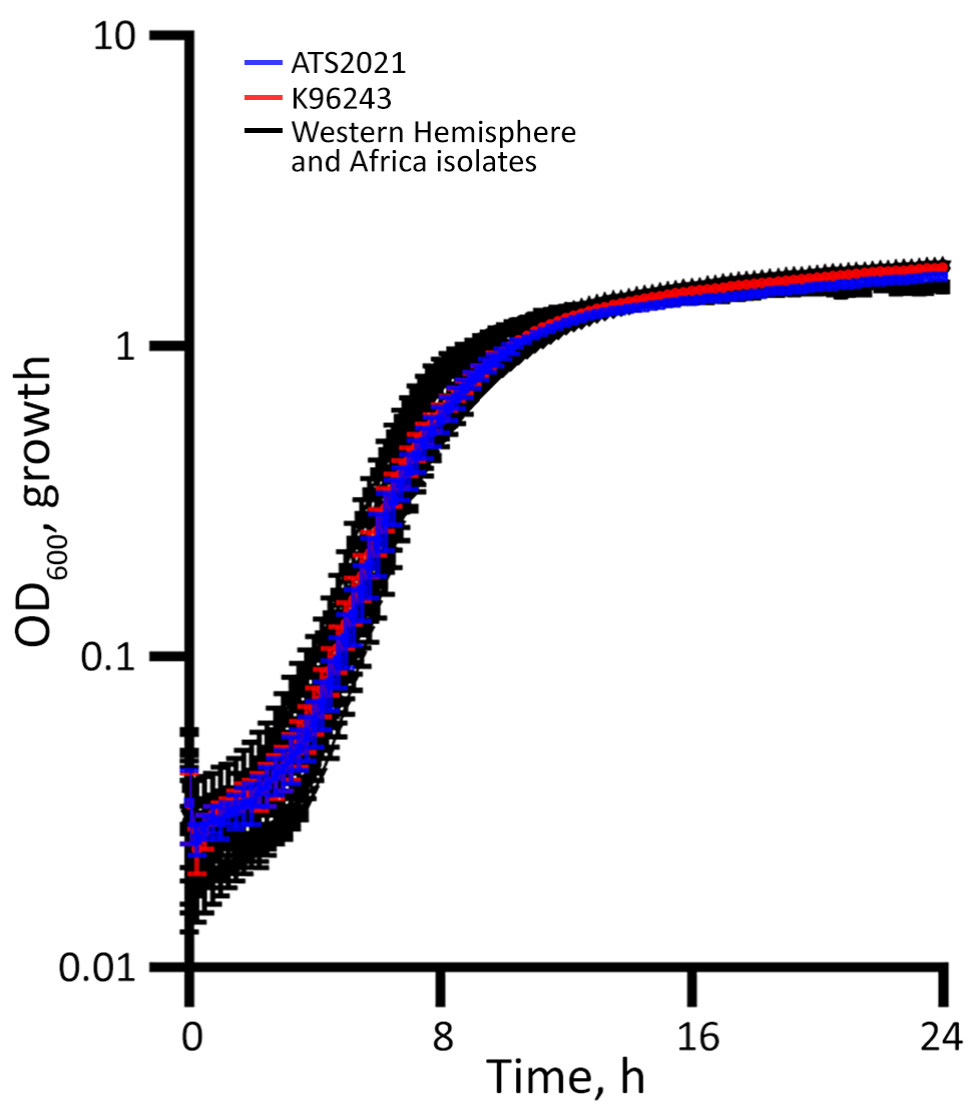
Western Hemisphere and Africa test panel isolates in a study of *Burkholderia pseudomallei* virulence in mouse models showing no differences in planktonic growth. *B. pseudomallei* was grown in Luria broth with 4% glycerol with shaking at 37°C over the course of 24 hours. We measured growth by optical density for >3 technical replicates in each experiment. Data displayed are the average results of 4 experiments. Error bars represent SE of the mean. OD_600_, optical density at 600 nm.

**Figure 3:**
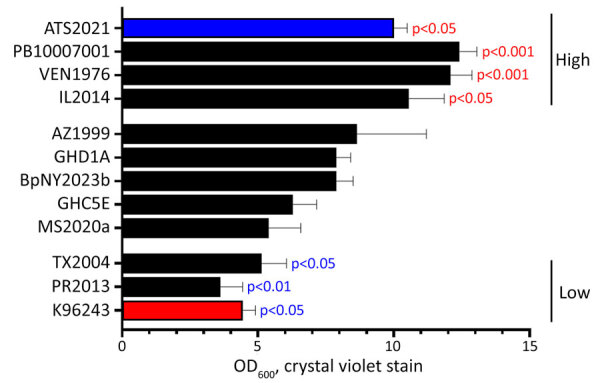
Observations of biofilm formation in a study of *Burkholderia pseudomallei* virulence in mouse models. *Burkholderia pseudomallei* isolates were inoculated into Luria broth with 4% glycerol and incubated statically at 37°C for 24 hours. We removed planktonic growth and stained the remaining biofilm by using crystal violet staining then quantified by using 600 nm optical density. We compared the test panel (represented by black bars) with a hyper-biofilm producing strain (strain ATS2021, represented by blue bar) and a common strain used in the laboratory with low biofilm formation (strain K96243, represented by red bar). Data displayed are the averaged results of >3 biologic replicates with error bars representing the standard error of the mean. Red p values indicate statistical significance relative to strain K96243. Blue p values indicate significance relative to strain ATS202. OD_600_, optical density at 600 nm.

We assembled a new panel of *B. pseudomallei* strains (Table 1) that are phylogeographically diverse. Our results underscore the phylogenetic diversity and geographic origin of the strains included in this study (Figure 1). We assessed the in vitro growth rates in LBG and identified near-identical growth rates for each isolate (Figure 2). In our evaluation of biofilm production, the different geographic isolates exhibited highly different biofilm production (Figure 3). We included 2 well-characterized strains, ATS2021 and K96243, for comparison. The ATS2021 strain was demonstrated as a hyper-biofilm producer (*26*), and the K96243 strain is one of the most used strains in melioidosis research.

**Table 1:** *Burkholderia pseudomallei* Western Hemisphere and African strain panel used in study of virulence of *B. pseudomallei* in mice*

Strain, NCBI SRA accession nos. (reference)	Location	Travel history	Clinical history	Collection type
GHD1A, SRR28096053 and SRR28096054 (*30*)	Ghana	NA	NA	Environmental, ST2058 novel strain type grouped within a subclade that include isolates from Burkina Faso
BpNY2023b, SRR35239810 (NA)	New York, USA	Ghana	27-year-old woman, cutaneous lesion on scalp	None
AZ1999, SRR9182277 and SRR9182846 (*12*)	Arizona, USA	El Salvador	37-year-old woman	None
VEN1976, SRR5221036 (*31*)	Venezuela	Unknown	Unknown	None
PB10007001, SRR5221028 (*31*)	Arizona, USA	Costa Rica	Woman, boat propeller wound to left thigh	None
IL2014, SRR5221014 (*32*)	Illinois, USA	Mexico	59-year-old woman, 4-day history of right-side, upper back, and anterior chest pain, dyspnea, and fevers. Diabetes mellitus, controlled HIV infection; cadaveric renal transplant recipient 13 months prior.	None
PR2013a, SRR5221018 (*31*)	Puerto Rico, USA	NA	NA	Environmental
GHC5E, SRR28096044 and SRR28096045 (*30*)	Ghana	NA	NA	Environmental, ST1749 similar Caribbean isolates
MS2020a, SRR23747754 (*11*)	Mississippi, USA	NA	39-year-old man, initial manifestation of acute respiratory distress and fever followed by multisystem organ failure. Admitted to intensive care unit with bilateral multilobar pneumonia and sepsis.	None
TX2004, SRR5221034 (*12*)	Texas, USA	Cryptic	82-year-old man with chronic renal insufficiency, diabetes, and hypertension. Cutaneous lesion on right hand.	Putative locally acquired

*NA, not available; NCBI SRA, National Center for Biotechnology Information Sequence Read Archive (https://www.ncbi.nlm.nih.gov/sra); ST, sequence type.

### In Vivo Median LD_50_ Estimations

**Figure 4:**
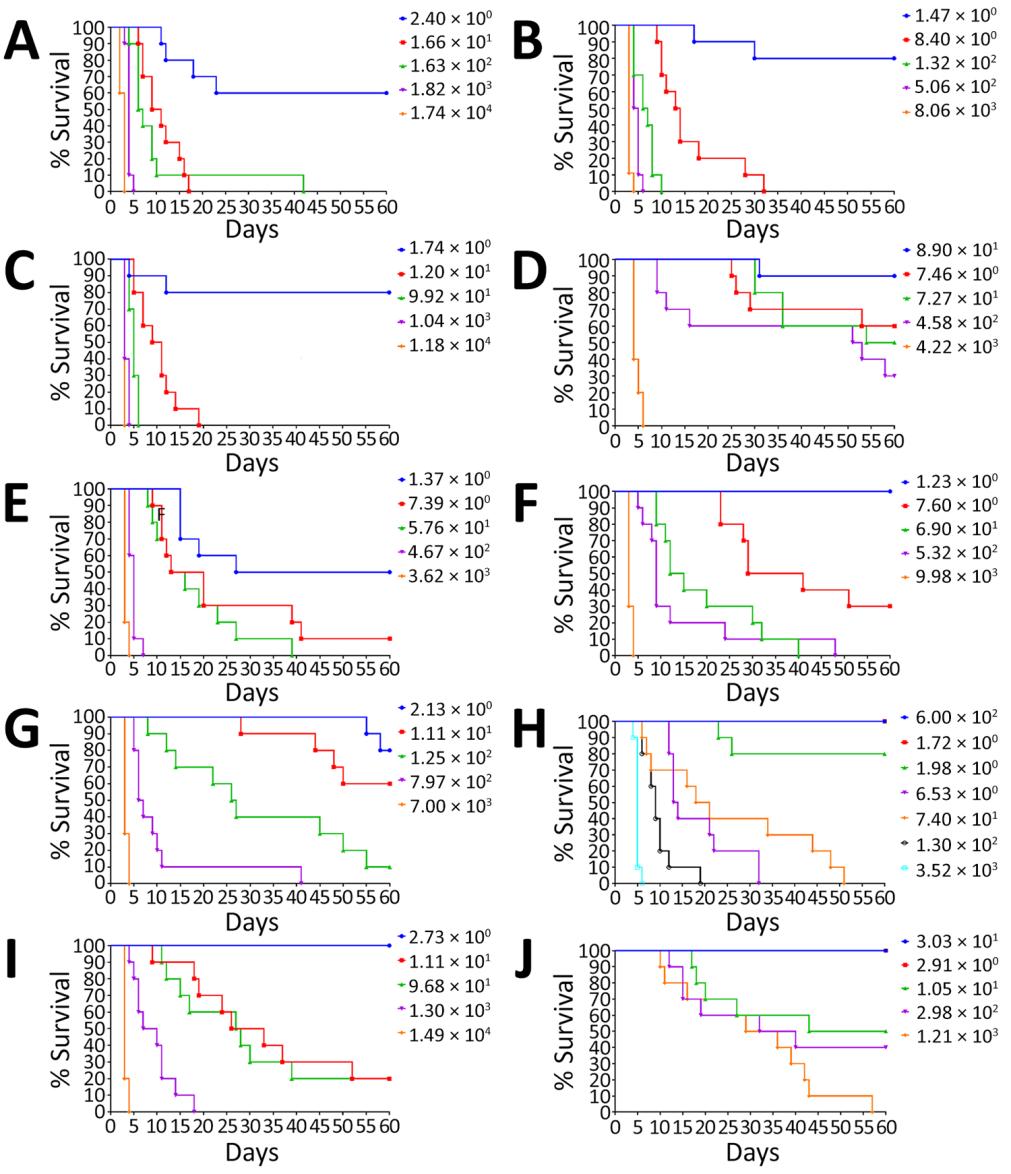
Survival rates for aerosolized challenge of Western Hemisphere and Africa *Burkholderia pseudomallei* strain virulence in BALB/c mouse models. We challenged BALB/c mice with 5 different doses of aerosolized *B. pseudomallei* isolates and observed them for 60 days (Appendix 1 Tables 1, 2). Each color represents a different dose. Isolates used for challenge A) GHD1A; B) PB10007001; C) BpNY2023b; D) IL2014; E) GHC5E; F) PR2013a; G) AZ1999; H) MS2020a; I) VEN1976; J) TX2004.

**Figure 5:**
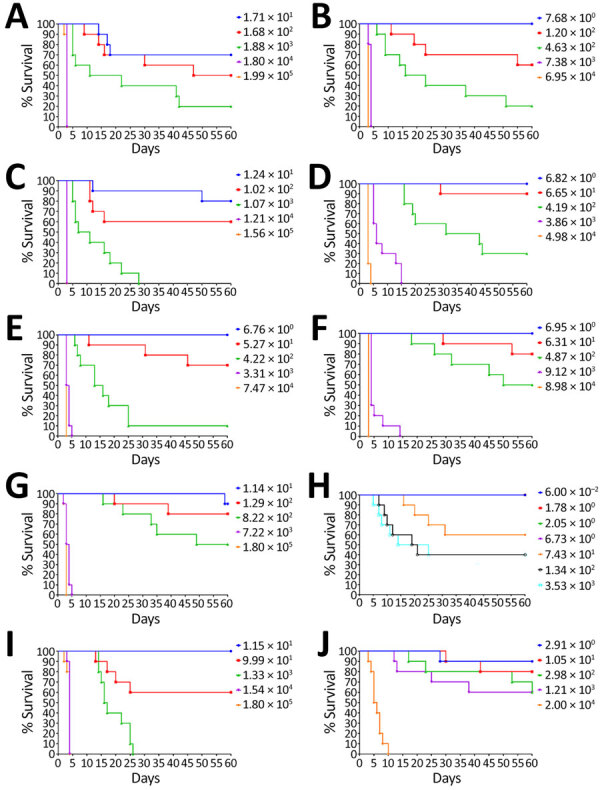
Survival rates for aerosolized challenge of Western Hemisphere and Africa *Burkholderia pseudomallei* strain virulence in C57BL/6 mouse models. C57BL/6 mice were challenged with 5 different doses of aerosolized *B. pseudomallei* isolates and observed for 60 days (Appendix 1 Tables 1, 2). Each color represents a different dose. Isolates used for challenge A) GHD1A; B) PB10007001; C) BpNY2023b; D) IL2014; E) GHC5E; F) PR2013a; G) AZ1999; H) MS2020a; I) VEN1976; J) TX2004.

We calculated LD_50_ values for each isolate in both BALB/c and C57BL/6 mice on day 21 and day 60 to help gauge the infection lethality during the first several weeks postexposure (acute phase) compared with the lethality observed throughout the entire 60-day study (Table 2; Figures 4, 5; Appendix 1 Tables 1, 2). The first pattern we observed from the dataset was that the 3 *B. pseudomallei* strains from Ghana (GHD1A, BpNY2023b, and GHC5E) displayed low LD_50_ estimates with rapid disease (day 21, <5 CFU/mL for BALB/c mice, 160–360 CFU/mL for C57BL/6 mice) and were among the most acutely lethal strains in the mouse model of inhalational melioidosis (day 60, 1–3 CFU/mL for BALB/c mice, 79–113 CFU/mL for C57BL/6 mice). The *B. pseudomallei* strain from Costa Rica (PB10007001) was approximately equally virulent when LD_50_ was compared with strains from Ghana (p>0.05). Second, the *B. pseudomallei* strains tested from Venezuela (VEN1976), Puerto Rico (PR2013a), and Mississippi (MS2020a) appeared less virulent during the first few weeks but continued to cause death through the 60-day study period, ultimately demonstrating major virulence by the study end. Last, relative to the other *B. pseudomallei* strains in this panel, the strains from Texas (TX2004), El Salvador (AZ1999), and Mexico (IL2014) exhibited slowly progressive virulence or displayed persistence-associated phenotypes at both the day 21 and day 60 postexposure to aerosolized bacteria. Of note, unlike the other *B. pseudomallei* strains tested, the strain from Mexico (IL2014) did not exhibit expected differential virulence in the 2 mouse strains. Instead, the LD_50_ estimates for IL2014 were virtually identical on day 21 (ratio of LD_50_ of 0.9; p = 0.87); although that ratio did increase to 6.3 (p = 0.0045) by the end of the 60-day study, the day 21 data are noteworthy (Table 2; Appendix 1 Table 1). Furthermore, analysis of TTD or euthanasia among all dose-groups identified that those 3 *B. pseudomallei* strains also had protracted TTD estimates. For example, at the completion of the study, the TX2004 strain had a median TTD of 32.5 (*10*,*33*) days in BALB/c mice for the highest dose evaluated, whereas all other strains had a median TTD of <5 at the highest dose tested. Similarly, strains AZ1999, IL2014, MS2020a, and VEN1976 offered additional examples of protracted TTD values (Appendix 1 Table 2).

**Table 2:** LD_50_ estimations in BALB/c and C57BL/6 mice calculated on day 21 and day 60 post exposure to aerosolized *Burkholderia pseudomallei* strains from the Western Hemisphere and Africa*

Strain	BALB/c mice 21-d LD_50_	C57BL/6 mice 21-d LD_50_	BALB/c mice 60-d LD_50_	C57BL/6 mice 60-d LD_50_
GHD1A	3.5 (0.2–10.5)	359.5 (65.9–1,487.9)	2.6 (2.4–16.6)†	113.4 (16.1–414.1)
BpNY2023b	2.1 (1.7–12.0)†	166.9 (52.4–515.1)	2.1 (1.7–12.0)†	79.4 (27.4–239.3)
AZ1999	215.4 (84.8–471.2)	1,420.3 (822.1–7,224.2)†	12.8 (4.2–35.6)	438.7 (150.4–1,418.8)
VEN1976	67.3 (26.2–217.5)	470.8 (155.8–1,485.2)	12.1 (11.1–96.8)†	108.2 (99.9–1,342.8)†
PB10007001	4.2 (2.1–8.9)	396.1 (178.1–1,445.4)	1.8 (1.5–8.4)†	169.6 (44.2–383.7)
IL2014	490.6 (458.1–4,223.7)†	448.6 (419.0–3,863.0)†	38.6 (8.5–155.1)	242.5 (111.6–581.6)
PR2013a	73.9 (26.9–190.8)	757.1 (486.6–9,123.9)†	6.7 (1.2–7.6)†	345.1 (132.7–1,172.8)
GHC5E	3.0 (0.1–11.1)	223.8 (97.1–526.2)	1.4 (0.0–3.1)	99.3 (42.3–228.8)
MS2020a	14.7 (0.4–3,490.4)	889.0 (235.6–13,773.1)	4.5 (3.1–9.1)	400.9 (125.5–2,708.2)
TX2004	6,869.3 (1,210.1–NC)†	2,124.6 (1,004.3–8,201.9)	88.0 (10.5–298.3)†	580.6 (134.6–4,314.3)

*Values are LD_50_ (95% CI). LD_50_, 50% lethal dose; NC, not calculated. †Because of the lack of response gradient across the tested doses, the model failed to provide confidence intervals for the LD_50_ estimate. However, we calculated LD_50_ confidence intervals on the basis of observed response rates,

### Determination of Residual Bacteria

At the end of the study (day 60), to identify potential unique bacterial dissemination patterns or evidence of pronounced chronicity in any of the uncharacterized *B. pseudomallei* strains, we evaluated the bacterial content of the lungs, spleens, and brains from surviving mice in select dose-groups. Our results (Tables 3, 4) were not entirely unexpected on the basis of previous *B. pseudomallei* panels (*28*,*34*), but we identified several trends of note. In the BALB/c mice, only the *B. pseudomallei* isolates that caused a slower progressing disease (AZ1999, IL2014, and TX2004) resulted in residual bacterial counts at the end of the 60-day study. *B. pseudomallei* strain PR2013a was an exception with the lowest dose examined (Table 3). As expected, the C57BL/6 mouse model had many more mice harboring bacteria at the end of the 60-day study (Table 4). Nearly every strain and dose we sampled at the end of study had evidence of bacteria in the brains of C57BL/6 mice, and more of the brains remained colonized compared with spleens. Although there are clear anatomic differences between mice and humans that could lead to artifactual bacterial pathogenesis, this prevalence of brain colonization should be considered a critical aspect of this model that could offer a mechanism to evaluate the effectiveness of medical countermeasures against neurologic melioidosis.

**Table 3:** Bacterial count in survivors of select dose-groups of BALB/c mouse models in a study of *Burkholderia pseudomallei* virulence

Strain	Inhaled dose	Organ	No. survivors	No. organs infected	CFU/g, range
GHD1A	2.40 × 10°	Lungs	6	0	0
		Spleen	6	0	0
		Brain	6	0	0
BpNY2003	1.74 × 10°	Lungs	8	0	0
		Spleen	8	0	0
		Brain	8	0	0
AZ1999	1.11 × 10^1^	Lungs	6	1	3.80 × 10^3^
		Spleen	6	1	1.25 × 10^4^
		Brain	6	0	0
VEN1976	1.11 × 10^1^	Lungs	2	0	0
		Spleen	2	0	0
		Brain	2	0	0
	9.68 × 10^1^	Lungs	2	0	0
		Spleen	2	0	0
		Brain	2	0	0
PB10007001	1.47 × 10°	Lungs	8	0	0
		Spleen	8	0	0
		Brain	8	0	0
IL2014	7.27 × 10^1^	Lungs	5	0	0
		Spleen	5	1	5.53 × 10^7^
		Brain	5	3	1.82 × 10^1^–3.02 × 10^2^
	4.58 × 10^2^	Lungs	3	2	3.13 × 10^1^–3.23 × 10^1^
		Spleen	3	3	1.13 × 10^6^–7.08 × 10^7^
		Brain	3	3	1.43 × 10^2^–5.54 × 10^3^
PR2013a	7.60 × 10°	Lungs	3	2	1.19 × 10^2^–9.79 × 10^2^
		Spleen	3	0	0
		Brain	3	0	0
GHC5E	1.37 × 10°	Lungs	5	0	0
		Spleen	5	0	0
		Brain	5	0	0
	7.39 × 10°	Lungs	1	0	0
		Spleen	1	0	0
		Brain	1	0	0
MS2020a	1.72 × 10^0^	Lungs	3	0	0
		Spleen	10	0	0
		Brain	3	0	0
	1.98 × 10^0^	Lungs	3	0	0
		Spleen	8	0	0
		Brain	3	0	0
TX2004	1.05 × 10^1^	Lungs	5	0	0
		Spleen	5	0	0
		Brain	5	0	0
	2.98 × 10^2^	Lungs	4	0	0
		Spleen	4	1	7.95 × 10^2^
		Brain	4	0	0

*CFU, colony forming units.

**Table 4:** Bacterial count in survivors of select dose-groups of C57BL/6 mouse models in a study of *Burkholderia pseudomallei* virulence

Strain	Inhaled dose	Organ	No. survivors	No. organs Infected	CFU/g, range
GHD1A	1.71 × 10^1^	Lungs	7	0	0
		Spleen	7	0	0
		Brain	7	1	9.26 × 10°
	1.68 × 10^2^	Lungs	5	2	3.18 × 10^3^–4.00 × 10^3^
		Spleen	5	0	0
		Brain	5	2	8.75 × 10^2^–2.19 × 10^3^
	1.88 × 10^3^	Lungs	2	1	2.94 × 10^1^
		Spleen	2	0	0
		Brain	2	1	8.93 × 10^1^
BpNY2023b	1.24 × 10^1^	Lungs	8	0	0
		Spleen	8	0	0
		Brain	8	1	1.54 × 10^1^
	1.02 × 10^2^	Lungs	5	1	2.26 × 10^3^
		Spleen	5	1	1.85 × 10^5^
		Brain	5	3	1.96 × 10^1^–3.93 × 10^2^
AZ1999	1.29 × 10^2^	Lungs	8	2	2.94 × 10^1^–1.07 × 10^2^
		Spleen	8	0	0
		Brain	8	6	1.00 × 10^1^–1.75 × 10^3^
	8.22 × 10^2^	Lungs	5	2	3.33 × 10^1^–1.45 × 10^2^
		Spleen	5	0	0
		Brain	5	5	1.31 × 10^2^–6.57 × 10^2^
VEN1976	9.99 × 10^1^	Lungs	6	1	7.21 × 10^2^
		Spleen	6	0	0
		Brain	6	1	1.44 × 10^3^
PB10007001	1.20 × 10^2^	Lungs	6	1	4.09 × 10^2^
		Spleen	6	0	0
		Brain	6	1	3.73 × 10^3^
	4.63 × 10^2^	Lungs	2	1	2.30 × 10^2^
		Spleen	2	0	0
		Brain	2	2	6.78 × 10^1^–6.76 × 10^2^
IL2014	6.65 × 10^1^	Lungs	9	1	1.07 × 10^4^
		Spleen	9	2	8.44 × 10^7^–1.08 × 10^8^
		Brain	9	6	7.41 × 10^1^–8.25 × 10^4^
	4.19 × 10^2^	Lungs	3	1	3.75 × 10^1^
		Spleen	3	0	0
		Brain	3	2	1.79 × 10^1^–2.19 × 10^2^
PR2013a	6.31 × 10^1^	Lungs	8	1	4.41 × 10^1^
		Spleen	8	0	0
		Brain	8	2	8.93 × 10^0^–3.23 × 10^1^
	4.87 × 10^2^	Lungs	5	1	4.00 × 10^1^
		Spleen	5	1	6.82 × 10^1^
		Brain	5	4	2.42 × 10^2^–2.75 × 10^3^
GHC5E	5.27 × 10^1^	Lungs	7	1	1.85 × 10^2^
		Spleen	7	0	0
		Brain	7	2	1.53 × 10^2^–3.51 × 10^2^
	4.22 × 10^2^	Lungs	1	0	0
		Spleen	1	0	0
		Brain	1	1	1.09 × 10^2^
MS2020a	7.43 × 10^1^	Lungs	3	0	0
		Spleen	6	0	0
		Brain	3	0	0
	3.53 × 10^3^	Lungs	4	1	Detected, unquantifiable because contaminated
		Spleen	4	1	Too numerous to count
		Brain	4	1	Detected, unquantifiable because contaminated
Tx2004	2.98 × 10^2^	Lungs	7	0	0
		Spleen	7	0	0
		Brain	7	0	0
	1.21 × 10^3^	Lungs	6	1	5.29 × 10^7^
		Spleen	6	1	1.20 × 10^9^
		Brain	6	1	1.25 × 10^2^

### Genome Analyses 

**Figure 6:**
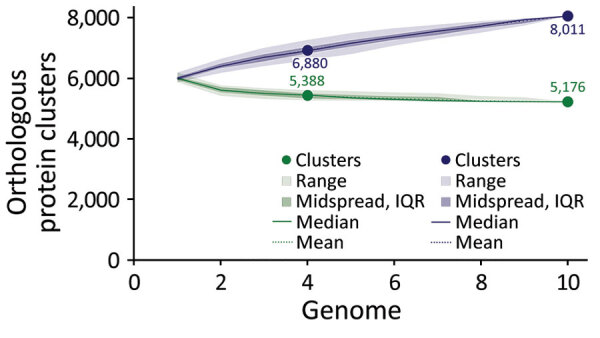
Pangenome analysis of *Burkholderia pseudomallei* strains from the Western Hemisphere and Africa in mouse models. We used panaroo version 1.5.2 (https://github.com/gtonkinhill/panaroo) for analysis of all 10 isolate genomes. The core genome size is 5,176 genes, and scoary version 1.6.16 (https://github.com/AdmiralenOla/Scoary) revealed 2 hypothetical protein encoding genes unique to the 3 virulent strains from Africa (GHD1A, GHC5E, BpNY2023b): an 84-residue encoding gene and a 262-residue encoding gene. The range, spread, median, and mean came from 100 permutations simulating genome addition and was plotted in R version 4.5.1 (The R Project for Statistical Computing, https://www.r-project.org) with refinements in inkscape v1.0.1 (https://inkscape.org). Purple represents pangenome; green represents core genome. IQR, interquartile range.

We conducted pangenome analysis of all 10 isolate genomes included in this study to identify gene candidates that could potentially account for the increased virulence associated with the 3 strains of African origin (GHD1A, GHC5E, and BpNY2023b). Whole-genome sequence accession numbers are included (Table 1). The core genome was determined to consist of 5,176 genes, whereas the pangenome was determined to consist of 8,011 total genes (Figure 6). We then further investigated the pangenome and identified genes unique to GHD1A, GHC5E, and BpNY2023b, finding 2 predicted hypothetical protein-encoding genes. Further motif queries of those 2 predicted protein-encoding genes suggest one 753-bp gene makes a transcriptional regulator and the other appears to be a truncated (255-bp) kumamolysin protease.

## Discussion

Our results are a comprehensive evaluation of virulence assessed in mouse models of inhalational melioidosis for strains of *B. pseudomallei* originating in the Western Hemisphere and Africa. The *B. pseudomallei* strains we evaluated offer a spectrum of virulence patterns; the Ghana strains uniformly produced acutely lethal disease despite unique genotypes and years between isolation (*30*,*35*). Our genomic analyses did not identify clear functional differences at the gene level that definitively distinguish among those strains of *B. pseudomallei.*

Of note, this study evaluated virulence in mice after exposure to small particle aerosols; however, it does not address virulence of the novel *B. pseudomallei* strains after ingestion or inoculation via parenteral routes. We did not observe any in vitro growth differences between the strains in the new test panel and the growth kinetics are remarkably similar to previously reported for strains originating in Thailand and northern Australia (*34*). Previously, when using a panel of *B. pseudomallei* strains originating from Australia and Thailand, we described a significant inverse correlation with biofilm production and virulence when assessed in mouse models of inhalational melioidosis (*26*). However, with this current panel of strains, we did not observe this correlation following the same challenge method. More work is required to ascertain if the correlation with inhalation virulence of *B. pseudomallei* is biologically relevant or if it is only associated with strains from Thailand or Northern Australia.

The role of the *B. pseudomallei* biofilm has been debated (*26*,*36*). However, typically biofilm is considered a critical virulence factor because it enables the bacterial community to withstand the hostile environment within a host and resist antimicrobial drugs (*37*). For instance, in vitro biofilm formation was found to be higher for isolates obtained from patients with relapsing melioidosis compared with isolates of patients without relapse (*38*). Further, biofilm forming capacity was implicated in antimicrobial drug susceptibility, although upregulation of biofilm-related genes might play a larger role in antimicrobial drug resistance than the biofilm matrix in *B. pseudomallei* (*33*,*39*). 

This analysis of *B. pseudomallei* strain virulence observed in strains from the Western hemisphere and Africa resulted in similar observations to those reported when examining strains from Thailand and northern Australia (*28*,*34*). Some strains are rapidly fatal in the mouse model, and the LD_50_ estimate remains similar throughout the course of the 60-day study (i.e., GHD1A, BpNY2023b, PB10007001, and GHC5). In contrast, other strains cause disease but require additional time to result in lethality (i.e., AZ1999, VEN1976, PR2013a, and MS2020a); 1 strain (IL2014) had very similar LD_50_ estimates at day 21 in both BALB/c and C57BL/6 mice. More work is required to understand the similar virulence in both mouse models seen with strain IL2014, but those findings mirror what we have previously described regarding the virulence of strain 1106a from Thailand (*28*,*40*). Those unique virulence patterns will be necessary to better characterize the mouse models of inhalational melioidosis and host immunity to this disease.

In our geographically diverse panel, we did not identify patterns that suggested differential virulence associated with isolate origin (i.e., clinical vs. environmental isolate), but we note that these differences have been documented (*41*,*42*). For example, when we compared the virulence of the 3 isolates from Ghana (2 environmental isolates with different genomic signatures and 1 human clinical isolate from 2023), we calculated similar median lethal dose estimates determined by the mouse in vivo models (p>0.22 in all cases). *B. pseudomallei* genomic analyses are complex, and additional analyses will be required to further identify genomic features that could lead to the classification of geographic isolates on the basis of virulence (*40*,*43*).

Virulence is difficult to define in *B. pseudomallei*. Complex acute clinical signs coupled with poorly characterized chronic or latent stages make melioidosis challenging to diagnose in humans and difficult to model in laboratory animals. We observed mice with inhalational melioidosis for 60 days, and in our estimation, that prolonged duration still might not be adequate to capture the unique disease phenotypes exhibited in those animals. Several examples of human case studies question the description of *B. pseudomallei* infection staying completely latent or dormant for decades, as opposed to the established pattern of relapsing or remitting chronic infection (*12*,*44*–*46*). Of interest, several of those examples include residents of the Gulf Coast region of the United States. Recently, an investigation estimated that activation from a truly asymptomatic infection represented <1.7%–2.9% of cases (*47*). Improved data collection and analyses, the reevaluation of seminal melioidosis case reports, and the growing dataset of *B. pseudomallei* in parts of the world outside of northern Australia and Southeast Asia further supports a much larger effect of melioidosis globally than previously estimated and underscores the complexities of melioidosis modeling or virulence assessment.

Our intent is not to oversimplify virulence attributes associated with *B. pseudomallei* isolates solely by rank ordering median lethal doses in mouse models. Understanding the virulence of this organism will require in-depth and comprehensive studies designed to examine macrophage infection assays, intracellular survival or replication assessments, resistance to host killing, and serial sampling natural history studies in appropriate animal models of disease. When taken together, those data would then offer a more complete view of virulence, particularly in context of other critical parameters that include but are not limited to host-inflammatory response, tissue pathology, bacterial burden or dissemination, and tissue tropisms that might be associated with isolates (*48*,*49*). Nevertheless, this study is a comprehensive attempt to begin to understand the virulence associated with Western Hemisphere and African isolates of *B. pseudomallei*. Of interest is the residual levels of bacteria in the brains of mice (Tables 3, 4). Every strain examined could be found in the brains of the C57BL/6 mice exposed to aerosolized *B. pseudomallei* (Table 4). Those data reinforce that the C57BL/6 model is less acute than the BALB/c model, likely enabling differential bacterial dissemination and replication in tissues such as the brain. Neurologic impacts are a devastating aspect of inhalational melioidosis; a better understanding of such neuropathogenesis and model refinement could have massive implications for modeling neurologic melioidosis (*50*; Appendix 2 reference *51*).

Although already a regulated biologic select agent because of biodefense concerns, *B. pseudomallei* is considered an emerging threat in the United States after being declared endemic to the Gulf Coast region (*11*). Historically, melioidosis has been attributed to travel outside of the continental United States, previously residing in an endemic part of the world, or rare exposure to imported products or a single case associated with an aquarium (*32*; Appendix 2 references *52–54*). The recent lethal cases of melioidosis associated with the unintentional importation of *B. pseudomallei* in aromatherapy spray manufactured in India were other examples of domestic melioidosis from an unexpected source (*18*,*26*). As we continue to understand the risk associated with understudied Western Hemisphere and African strains, it is imperative to perform additional studies. Those data are necessary for medical modeling purposes, risk assessments, and to further educate medical providers in areas of the world where *B. pseudomallei* is not an expected pathogen (Appendix 2 reference *55*).

In conclusion, melioidosis is a challenging disease to study because of its tremendously diverse clinical manifestations in both human patients and laboratory animal models. For the best possible patient outcome, it is vital that this disease is accurately diagnosed and appropriate antimicrobial drug treatments are provided (Appendix 2 references 56,57). In addition to being an emerging threat globally, melioidosis also offers an excellent example of the necessity of continuing research into infectious diseases and challenging accepted dogma as new data become available. The concept that pathogens can be studied and treated with effective novel or repurposed medical countermeasures but research on the organism abandoned because of the treatment successes is counterproductive to both biodefense and public health fields of research. We must continue to monitor biothreats, reexamine risk assessments and disease modeling efforts, and classify or reclassify emerging and reemerging threats to be optimally prepared for future adversarial use, outbreaks, or pandemics.

Appendix 1 Additional information about virulence of *Burkholderia pseudomallei* strains from Western Hemisphere and Africa in mice.

Appendix 2 Additional references providing background information about the virulence of *Burkholderia pseudomallei* strains from Western Hemisphere and Africa in mice.
