# Supplementary material for: Virulence of Burkholderia pseudomallei Strains from Western Hemisphere and Africa in Mice
Source: Emerg Infect Dis. 2026 Aug;32(8):1251–63. doi: 10.3201/eid3208.260069 (PMC13426869; doi:10.3201/eid3208.260069)
Supplement: Appendix 2 — Additional references providing background information about the virulence of Burkholderia pseudomallei strains from Western Hemisphere and Africa in mice. [file 26-0069-Techapp-s2.pdf]

Article DOI: https://doi.org/10.3201/eid3208.260069

EID cannot ensure accessibility for supplementary materials supplied by authors. Readers who have difficulty accessing supplementary content should contact the authors for assistance.

Virulence of *Burkholderia pseudomallei* Strains from Western Hemisphere and Africa in Mice

Appendix 2

Appendix References

51. Currie BJ, Fisher DA, Howard DM, Burrow JN. Neurological melioidosis. Acta Trop. 2000;74:145–51. PubMed https://doi.org/10.1016/S0001-706X(99)00064-9

52. Zehnder AM, Hawkins MG, Koski MA, Lifland B, Byrne BA, Swanson AA, et al. *Burkholderia pseudomallei* isolates in 2 pet iguanas, California, USA. Emerg Infect Dis. 2014;20:304–6. PubMed https://doi.org/10.3201/eid2002.131314

53. Dawson P, Duwell MM, Elrod MG, Thompson RJ, Crum DA, Jacobs RM, et al. Human melioidosis caused by novel transmission of *Burkholderia pseudomallei* from freshwater home aquarium, United States. Emerg Infect Dis. 2021;27:3030–5. PubMed https://doi.org/10.3201/eid2712.211756

54. Currie BJ. Melioidosis: an important cause of pneumonia in residents of and travellers returned from endemic regions. Eur Respir J. 2003;22:542–50. PubMed https://doi.org/10.1183/09031936.03.00006203

55. Torres AG. The public health significance of finding autochthonous melioidosis cases in the continental United States. PLoS Negl Trop Dis. 2023;17:e0011550. PubMed https://doi.org/10.1371/journal.pntd.0011550

56. Currie BJ, Mayo M, Ward LM, Kaestli M, Meumann EM, Webb JR, et al. The Darwin prospective melioidosis study: a 30-year prospective, observational investigation. Lancet Infect Dis. 2021;21:1737–46. PubMed https://doi.org/10.1016/S1473-3099(21)00022-0

57. Sullivan RP, Marshall CS, Anstey NM, Ward L, Currie BJ. 2020 Review and revision of the 2015 Darwin melioidosis treatment guideline; paradigm drift not shift. PLoS Negl Trop Dis. 2020;14:e0008659. PubMed https://doi.org/10.1371/journal.pntd.0008659
